# Screening of Bacterial Vaginosis in Antenatal Women Between 18 and 24 Weeks of Gestational Age and Its Association With Preterm Premature Rupture of Membranes, Preterm Labour, and Early Neonatal Outcomes: A Prospective Observational Cohort Study

**DOI:** 10.7759/cureus.110709

**Published:** 2026-06-12

**Authors:** Shalini Mukherjee, Jyochnamayi Panda, Priyanka Deshmukh, Niharika Pathak

**Affiliations:** 1 Obstetrics and Gynaecology, Kalinga Institute of Medical Sciences, Bhubaneswar, IND; 2 Obstetrics and Gynaecology, Kalinga Institute of Medical Sciences, Bhubaneshwar, IND

**Keywords:** bacterial vaginosis, nugent score, pprom, pregnancy outcomes, preterm labour

## Abstract

Background

Bacterial vaginosis (BV) is the most common lower genital tract infection among women of reproductive age and has been consistently associated with adverse pregnancy outcomes. During pregnancy, disruption of the normal *Lactobacillus*-dominant vaginal flora may predispose women to ascending infections, increasing the risk of preterm premature rupture of membranes (PPROM) and preterm labour (PTL).

Objective

The present study aimed to assess the prevalence of bacterial vaginosis among antenatal women between 18 and 24 weeks of gestation and to evaluate its association with preterm premature rupture of membranes (PPROM), preterm labour (PTL), and early neonatal outcomes, including birth weight, Apgar scores at 1 and 5 minutes, and neonatal ICU (NICU) admission by estimating relative risks (RRs) and corresponding 95% confidence intervals for adverse maternal and neonatal outcomes. Additionally, the study aims to explore the potential clinical relevance of bacterial vaginosis as a risk marker for adverse pregnancy outcomes within the context of an observational cohort design.

Methods

This prospective observational cohort study included 220 antenatal women with singleton pregnancies between 18 and 24 weeks of gestation attending a tertiary care hospital. Participants were screened for BV using vaginal pH testing, whiff test, wet mount microscopy, Gram staining, and Nugent scoring. Women were followed until delivery, and maternal outcomes (PPROM, PTL, gestational age at delivery) and neonatal outcomes (birth weight, Apgar scores, NICU admission) were recorded. Statistical analysis was performed using Student’s t-test and Chi-square/Fisher’s exact test, with a p-value <0.05 considered statistically significant. The study was conducted from May 2023 to April 2025, and statistical analysis was performed using SPSS version 25.0 (IBM Corp., Armonk, USA).

Results

The prevalence of BV was 110/220 (50%). PPROM occurred in 27/110 (24.5%) of BV-positive women compared to 3.6% of BV-negative women (RR = 6.8; p < 0.001). Preterm labour was observed in 23/110 (20.9%) of BV-positive women versus 4/110 (3.6%) in BV-negative women (RR = 5.8; p < 0.001). BV-positive women delivered at a significantly lower mean gestational age. Neonates born to BV-positive mothers had significantly higher rates of low Apgar scores at 1 minute, 86/110 (78.2%) vs 61/110 (55.5%), and 5 minutes, 48/110 (43.6%) vs 20/110 (18.2%), p < 0.001.

Conclusions

Bacterial vaginosis was highly prevalent in mid-pregnancy and showed a strong association with PPROM, PTL, and adverse early neonatal outcomes. Routine screening for BV between 18 and 24 weeks of gestation may help identify women at risk and potentially reduce preterm birth-related morbidity.

## Introduction

Bacterial vaginosis (BV) is characterized by a shift from normal *Lactobacillus*-dominant vaginal flora to a polymicrobial overgrowth of anaerobic organisms such as *Gardnerella vaginalis*, *Mobiluncus *species, and *Prevotella*. This alteration leads to elevated vaginal pH, depletion of protective lactobacilli, and the presence of clue cells, forming the basis of commonly used diagnostic criteria such as the Amselcriteria and Nugent scoring [1,2].

During pregnancy, BV assumes particular clinical importance because of its association with adverse outcomes, including preterm premature rupture of membranes (PPROM), preterm labour (PTL), low birth weight, chorioamnionitis, and neonatal sepsis [2-4]. Ascending infection from the lower genital tract can trigger inflammatory cascades that weaken fetal membranes and stimulate uterine contractions, leading to premature delivery [3,4].

Management during pregnancy usually involves metronidazole or clindamycin, although the effectiveness of treatment in preventing preterm birth varies across studies [5].

Globally, the prevalence of bacterial vaginosis among women of reproductive age ranges from 20% to 50%, with higher rates reported in low- and middle-income countries [6,7]. Indian studies have reported a prevalence of 25-40% among pregnant women [8-11], and urogenital infections, including BV, have been identified as significant contributors to preterm labour in Indian populations [9].

Bacterial vaginosis is clinically characterized by thin homogeneous vaginal discharge, fishy odour, elevated vaginal pH, and the presence of clue cells [6]. Diagnostic methods include Amsel’s clinical criteria and Nugent scoring based on Gram-stained vaginal smears, the latter being considered the gold standard [1].

India bears a disproportionately high burden of preterm births, many of which are potentially preventable. Despite this, routine antenatal screening for BV is not universally practiced. Screening during mid-pregnancy (18-24 weeks) provides a critical window for early detection and intervention before irreversible inflammatory damage occurs. Ascending infection and inflammatory cytokine release associated with BV during this gestational period may lead to weakening of fetal membranes and premature activation of labour pathways [12,13]. Several studies have demonstrated a stronger association between BV diagnosed before 24 weeks of gestation and adverse pregnancy outcomes compared to BV detected later in pregnancy [3,14].

In addition to biological mechanisms, social and structural determinants of health play a crucial role in the occurrence and outcomes of bacterial vaginosis during pregnancy. Factors such as low socioeconomic status, limited access to antenatal care, poor genital hygiene, nutritional deficiencies, and variations in health-seeking behavior can influence both the prevalence of BV and the risk of adverse pregnancy outcomes, including preterm birth. In low- and middle-income settings, delayed diagnosis and inadequate treatment of genitourinary infections may further compound these risks. Recognizing these broader determinants is essential for a more comprehensive understanding of BV and its impact on maternal and neonatal health.

The present study aimed to assess the prevalence of BV among antenatal women between 18 and 24 weeks of gestation and examine its association with PPROM, PTL, and neonatal outcomes.

Neonatal outcomes evaluated in the present study included birth weight, Apgar scores at 1 and 5 minutes, and the requirement for neonatal ICU (NICU) admission.

## Materials and methods

A prospective observational cohort study was conducted in the Department of Obstetrics and Gynaecology at a tertiary care hospital from May 2023 to April 2025 after obtaining approval from the Institutional Ethics Committee (Approval No: KIIT/KIMS/1207/2023, dated 27 February 2023). Antenatal women were classified into two groups based on exposure status: those diagnosed with bacterial vaginosis (BV) between 18 and 24 weeks of gestation (exposed group) and those without BV (unexposed group). All participants were followed prospectively from the time of screening until delivery to assess maternal and neonatal outcomes.

A total of 220 antenatal women with singleton pregnancies between 18 and 24 weeks of gestation were enrolled after obtaining written informed consent. Women aged 18-40 years who were willing to participate were included in the study. Those with multiple gestations, recent antibiotic use within the preceding 14 days, active systemic infections, vaginal bleeding, cervical cerclage, or major fetal anomalies were excluded.

The study population consisted of antenatal women attending a tertiary care hospital who were consecutively recruited during routine antenatal visits between 18 and 24 weeks of gestation. This gestational window was selected as it represents a critical period for identifying asymptomatic infections prior to the onset of inflammatory pathways leading to preterm labour and PPROM.

Baseline data were collected through detailed history taking, including maternal age, parity, prior history of preterm birth or PPROM, previous genital tract infections, and any complications in the current pregnancy, followed by general and obstetric examination. Vaginal samples were obtained aseptically from the posterior fornix. Vaginal pH was assessed using indicator strips, with a value greater than 4.5 considered abnormal. The whiff test was performed using 10% potassium hydroxide. Wet mount microscopy was used to detect clue cells, yeast, and inflammatory cells. Additionally, Gram-stained smears were evaluated using the Nugent scoring system, which is widely regarded as the gold standard for the diagnosis of bacterial vaginosis [1]. Women who were positive for BV were considered the exposed cohort, while women who were negative for BV served as the unexposed cohort.

Participants were subsequently monitored until delivery for outcome assessment. Women diagnosed with bacterial vaginosis were managed according to standard institutional protocols. The primary outcomes included preterm premature rupture of membranes (PPROM), defined as rupture of membranes before 37 completed weeks of gestation prior to the onset of labour, and preterm labour (PTL), defined as the onset of labour before 37 completed weeks. Secondary outcomes included gestational age at delivery, birth weight, Apgar scores at 1 and 5 minutes, and the need for neonatal intensive care unit (NICU) admission.

Sample size calculation

Based on a previous study by Nguyen et al [15], taking the odds ratio of bacterial vaginosis (OR=3.16; 95% CI=1.23-8.15; p=0.016) for preterm labor, at a 5% level of significance, 95% confidence interval, and 95% power, the minimum calculated sample size was 220. Although the odds ratio was used for sample size estimation based on available literature, relative risk was used as the primary measure of association in this cohort study.

Statistical analysis

Continuous variables were expressed as mean ± standard deviation and compared using Student’s t-test. Categorical variables were expressed as frequencies and percentages and analyzed using the Chi-square test or Fisher’s exact test, as appropriate. Relative risk (RR) with 95% confidence intervals (CI) was calculated for dichotomous maternal and neonatal outcomes to estimate the strength of association between bacterial vaginosis and adverse pregnancy outcomes. RR was calculated by comparing the incidence of outcomes among BV-positive women with that among BV-negative women. A p-value <0.05 was considered statistically significant.

## Results

The demographic and baseline characteristics of the study participants are presented in Table 1. A total of 220 women were included, with 110 (50%) diagnosed with BV. The mean maternal age was comparable between groups (29.10 ± 5.03 vs 30.09 ± 4.71 years; p = 0.133, t = 1.51). The mean gestational age at screening was slightly lower in the BV-positive group (21.87 ± 1.23 weeks vs 22.17 ± 1.00 weeks; p = 0.046, t = 2.01). This finding suggests that BV-related microbial imbalance may occur earlier in mid-pregnancy, potentially allowing an opportunity for early identification and intervention. Other baseline parameters assessed included parity and obstetric history, which were comparable between groups.

**Table 1:** Demographic and baseline characteristics (N = 220) p < 0.05 is considered significant. Significant values are marked with (*). Tests used: Student’s t-test for continuous variables; Chi-square test/Fisher’s exact test for categorical variables BV: bacterial vaginosis

Variable	BV Absent (n=110)	BV Present (n=110)	Test Statistic	p-value
Age, years, mean ± SD	29.10 ± 5.03	30.09 ± 4.71	t = 1.51	0.133
Gestational age, weeks, mean ± SD	22.17 ± 1.00	21.87 ± 1.23	t = 2.01	0.046*

The diagnostic findings associated with bacterial vaginosis are summarized in Table 2. Women with BV had significantly higher vaginal pH (5.47 ± 0.67 vs 5.14 ± 0.68; p < 0.001, t = 3.89) and higher Nugent scores (6.61 ± 1.27 vs 2.50 ± 1.52; p < 0.001, t = 15.2). Clue cells were more frequently observed in BV-positive women (41/110 (37.3%) vs 13/110 (11.8%); χ² = 18.6, p < 0.001). Vaginal pH, clue cells, whiff test, and Nugent scoring were used as established diagnostic markers of BV as described in previous studies [1,2]. Women with BV were 2.5 times more likely to have a positive whiff test and 3.15 times more likely to have clue cells.

**Table 2:** Diagnostic findings of BV p < 0.05 is considered significant. Significant values are marked with (*). Tests used: Student’s t-test for continuous variables; Chi-square test/Fisher’s exact test for categorical variables. BV: bacterial vaginosis; RR: relative risk

Diagnostic Parameter	BV Absent (n=110)	BV Present (n=110)	Test Statistic	p-value	RR
Elevated pH, mean ± SD	5.14 ± 0.68	5.47 ± 0.67	t = 3.89	<0.001*	
Positive Whiff test, n/N (%)	2/110 (1.8%)	5/110 (4.5%)	χ² = 1.37	0.242	2.50
Clue cells present, n/N (%)	13/110 (11.8%)	41/110 (37.3%)	χ² = 18.6	<0.001*	3.15
Nugent Score, mean ± SD	2.50 ± 1.52	6.61 ± 1.27	t = 15.2	<0.001*	

The association between bacterial vaginosis and adverse pregnancy outcomes is shown in Table 3. PPROM occurred in 27/110 (24.5%) BV-positive women compared with 4/110 (3.6%) BV-negative women. BV-positive women had a significantly increased risk of PPROM (RR = 6.75; 95% CI: 2.45-18.58; p < 0.001). Preterm labour occurred in 23/110 (20.9%) BV-positive women compared with 4/110 (3.6%) BV-negative women. The risk of preterm labour was significantly higher among BV-positive women (RR = 5.75; 95% CI: 2.10-15.74; p < 0.001).

**Table 3:** Association of BV with PPROM and preterm labour p < 0.05 is considered significant. Significant values are marked with (*). Tests used: Student’s t-test for continuous variables; Chi-square test/Fisher’s exact test for categorical variables. BV: bacterial vaginosis; PPROM: preterm premature rupture of membranes

Outcome	BV Absent (n=110)	BV Present (n=110)	Relative Risk (95% CI)	Test Statistic	p-value
PPROM, n/N (%)	4/110 (3.6%)	27/110 (24.5%)	6.75 (2.45–18.58)	χ² = 21.8	<0.001*
Preterm Labour, n/N (%)	4/110 (3.6%)	23/110 (20.9%)	5.75 (2.10–15.74)	χ² = 17.9	<0.001*

Neonatal outcomes in relation to maternal BV status are presented in Table 4. Neonates born to BV-positive mothers had significantly lower Apgar scores at 1 minute (86/110 (78.2%) vs 61/110 (55.5%); χ² = 12.4, p < 0.001) and 5 minutes (48/110 (43.6%) vs 20/110 (18.2%); χ² = 15.3, p < 0.001). However, NICU admission rates were not significantly different (28/110 (25.5%) vs 40/110 (36.4%); p = 0.19). Compared with the BV-negative group, neonates born to BV-positive mothers were 1.41 times more likely to have an Apgar score <7 at 1 minute and 2.40 times more likely to have an Apgar score <7 at 5 minutes. In contrast, NICU admission was less frequent among neonates of BV-positive mothers (RR = 0.70); however, this association did not reach statistical significance (p = 0.19). Although Apgar scores were significantly lower, NICU admission depends on multiple clinical and institutional factors; therefore, not all neonates with transient low Apgar scores required NICU care.

**Table 4:** Neonatal outcomes in relation to BV p < 0.05 is considered significant. Significant values are marked with (*). Tests used: Student’s t-test for continuous variables; Chi-square test/Fisher’s exact test for categorical variables. BV: bacterial vaginosis

Neonatal Outcome	BV Absent (n=110)	BV Present (n=110)	Test Statistic	p-value	RR
Birth Weight (kg), mean ± SD	2.43 ± 0.54	2.36 ± 0.49	t = 1.04	0.298	
Apgar <7 at 1 min, n/N (%)	61/110 (55.5%)	86/110 (78.2%)	χ² = 12.4	<0.001*	1.41
Apgar <7 at 5 min, n/N (%)	20/110 (18.2%)	48/110 (43.6%)	χ² = 15.3	<0.001*	2.40
NICU Admission, n/N (%)	40/110 (36.4%)	28/110 (25.5%)	χ² = 2.32	0.19	0.70

## Discussion

Bacterial vaginosis (BV) continues to be a major reproductive health concern, particularly during pregnancy, where alterations in the vaginal microbiome have been strongly associated with adverse maternal and neonatal outcomes [15]. The present study, conducted among 220 antenatal women between 18 and 24 weeks of gestation, provides valuable insight into the prevalence of BV and its relationship with preterm premature rupture of membranes (PPROM), preterm labour (PTL), and neonatal wellbeing. By employing Nugent scoring-the gold standard for BV diagnosis-alongside established clinical markers such as vaginal pH, whiff test, and clue cells, the study offers a comprehensive assessment of microbial disturbance and its clinical consequences [1].

The prevalence of BV in the current study was 50%, which is notably higher than that reported in many Indian and international studies, where prevalence ranges from 20% to 40%. Studies by Madhivanan et al. and Verma et al. have reported BV prevalence between 25-32% among Indian pregnant women, while international datasets such as those from the Centers for Disease Control and Prevention report prevalence around 29% [6,7,9,13]. The elevated rate observed in the present study may reflect regional variations, socio-demographic factors, or differences in hygiene practices and sexual behavior. It may also suggest under-recognition of asymptomatic BV in routine antenatal care, emphasizing the importance of mid-pregnancy screening [6].

The diagnostic findings observed in this study are consistent with the established criteria for bacterial vaginosis. Women diagnosed with BV demonstrated significantly higher vaginal pH, increased presence of clue cells, and higher Nugent scores compared to those without BV. These findings reflect the underlying diagnostic framework used to identify BV rather than independent associations [1,2]. The higher Nugent scores observed among BV-positive women indicate a shift in vaginal flora, characterized by a relative reduction in *Lactobacillus *and an increase in anaerobic organisms; however, it is important to note that *Lactobacillus *levels were not directly measured in this study and are inferred from the scoring system [1,12]. These observations are in line with the conventional microbiological understanding of BV [1,12].

One of the most significant findings of this study is the strong association between BV and PPROM. This finding is consistent with previous studies [4,8]. PPROM occurred in a substantially higher proportion of BV-positive women compared to BV-negative women. Similar observations have been reported by Leitich et al., who demonstrated an increased risk of membrane rupture among women with BV [3]. The biological explanation for this association lies in the ability of BV-associated organisms to produce mucolytic enzymes and inflammatory cytokines, leading to degradation of collagen and weakening of fetal membranes [10,14]. Additionally, these pathogens stimulate matrix metalloproteinases, further increasing membrane susceptibility to rupture [3,4,8].

The association between BV and preterm labour observed in this study is also consistent with existing literature [4,8]. Ascending infection from the lower genital tract results in decidual inflammation, increased prostaglandin synthesis, and premature cervical ripening, all of which contribute to the early onset of labour. These findings further support the role of BV as a modifiable risk factor for preterm birth, as demonstrated in previous studies [16]. Similar findings have been reported across different populations [9,10].

Neonatal outcomes in the present study further highlight the clinical relevance of bacterial vaginosis during pregnancy. Although mean birth weight did not differ significantly between groups, neonates born to BV-positive mothers had significantly lower Apgar scores at both 1 and 5 minutes. These findings may reflect impaired early neonatal adaptation, potentially related to intrauterine exposure to infection or subclinical inflammation. Similar associations with increased neonatal morbidity and respiratory compromise have been reported in previous studies [8,14,16-22].

However, NICU admission rates were not significantly different between the two groups. This underscores the multifactorial nature of NICU admission, which is influenced not only by neonatal condition at birth but also by institutional protocols and clinical decision-making. Furthermore, despite the observed differences in Apgar scores, many cases may have been transient and did not require intensive care. These findings emphasize the need for cautious interpretation of neonatal outcomes to avoid overestimating the clinical impact of maternal BV.

Another noteworthy finding was the reduced mean gestational age at delivery among women with BV. Even a modest reduction in gestational age can have important implications, particularly in late preterm infants who remain at increased risk of respiratory and neurological morbidity compared to term infants [2,4].

Screening at 18-24 weeks of gestation is particularly relevant, as this period allows early identification and timely intervention. Several clinical trials, including those by Ugwumadu et al., have demonstrated that treatment of BV during early or mid-pregnancy may reduce the risk of PPROM and PTL, although results have varied [4,5,11,17]. The present findings support the rationale for incorporating BV screening into routine antenatal care, particularly in high-risk populations [5,6,17]. The sample size calculation and prospective design further strengthen the validity of the observed associations.

It is important to note that, given the observational nature of the study, the associations identified do not imply causality. While bacterial vaginosis was significantly associated with adverse maternal and neonatal outcomes, a direct cause-and-effect relationship cannot be established

Although the associations between bacterial vaginosis and adverse outcomes such as PPROM and preterm labour were statistically significant and of considerable magnitude, these findings should be interpreted with caution. The absence of adjustment for potential confounding variables, including socioeconomic status, obstetric history, and other maternal risk factors, may have influenced the strength of these associations. Therefore, the observed relative risks may represent an overestimation of the true effect.

From a public health perspective, the findings of this study highlight the potential importance of early identification of bacterial vaginosis during pregnancy, particularly in low- and middle-income settings where the burden of preterm birth is high and access to advanced neonatal care may be limited. In such settings, preventive strategies, including targeted screening, timely diagnosis, and appropriate management of genitourinary infections, may play a crucial role in reducing adverse pregnancy outcomes. Strengthening antenatal care services and improving awareness regarding reproductive health and hygiene practices could further contribute to better maternal and neonatal outcomes.

The present study has certain limitations. First, the study population was derived from a single tertiary care center, which may limit generalizability. Second, potential confounding variables such as parity, prior obstetric history, socioeconomic status, educational level, and hygiene practices were not adjusted for, which may have influenced the observed associations. Third, multivariate analysis was not performed, limiting the ability to determine independent predictors of adverse outcomes. Additionally, treatment for bacterial vaginosis was not controlled for in the analysis, which could have modified pregnancy outcomes. 

## Conclusions

Bacterial vaginosis was found to be highly prevalent among antenatal women in mid-pregnancy and was significantly associated with preterm premature rupture of membranes and preterm labour. The observed associations with adverse neonatal outcomes, including lower Apgar scores and reduced gestational age at delivery, further underscore its potential clinical relevance. Routine screening for BV between 18 and 24 weeks of gestation may help identify women at higher risk and could contribute to improved maternal and neonatal outcomes, although further large-scale studies are warranted. However, given the observational nature of the study, these findings should be interpreted as associations rather than causal relationships. Additionally, the absence of adjustment for potential confounding variables may have influenced the strength of these associations. Further large-scale studies incorporating multivariate analyses and evaluating the impact of screening and treatment interventions are warranted to better define the role of bacterial vaginosis in adverse pregnancy outcomes.
